# The effects of the covid-19 pandemic on puberty: a cross-sectional, multicenter study from Turkey

**DOI:** 10.1186/s13052-022-01337-z

**Published:** 2022-08-13

**Authors:** Gul Yesiltepe Mutlu, Elif Eviz, Belma Haliloglu, Heves Kirmizibekmez, Fatma Dursun, Servan Ozalkak, Atilla Cayir, Beste Yuksel Sacli, Mehmet Nuri Ozbek, Huseyin Demirbilek, Sukru Hatun

**Affiliations:** 1 Department of Pediatric Endocrinology and Diabetes, Koç University School of Medicine, Koç Üniversitesi Hastanesi, Davutpaşa Cd. No:4, Topkapı, 34010 Turkey; 2 Department of Pediatric Endocrinology and Diabetes, Yeditepe University School of Medicine, Istanbul, Turkey; 3 Department of Pediatric Endocrinology, University of Health Sciences, Umraniye Training and Research Hospital, Istanbul, Turkey; 4 Clinic of Pediatric Endocrinology, University of Health Sciences Turkey, Diyarbakir Gazi Yasargil Training and Research Hospital, Diyarbakir, Turkey; 5 Department of Pediatric Endocrinology, Erzurum Regional Training and Research Hospital, Erzurum, Turkey; 6 Faculty of Medicine, Department of Pediatric Endocrinology, Hacettepe University, Ankara, Turkey

**Keywords:** Covid-19, Pandemic, Early puberty, Girls

## Abstract

**Backgrounds:**

During the Coronavirus-19 disease (Covid-19) pandemic it was observed that the number of girls presenting with early puberty had increased. The aim of this study was to carry out a retrospective evaluation of the characteristics of girls who had been referred for evaluation of precocious puberty in five different pediatric endocrinology units, before and during the pandemic.

**Methods:**

The study participants comprised 359 girls who were assigned into 2 groups a pre-pandemic group (*n*:214) and a pandemic group (*n*:145). Those participants (*n*:99) who had medical records in the follow-up period were classified into 3 subgroups according to the time of presentation and follow-up visits (group-1: first admission and follow-up visit before the pandemic, group-2: first admission before the pandemic, the follow-up visit during the pandemic, group-3: first admission and follow-up visit during the pandemic).

**Results:**

The age at presentation and age at pubertal onset were both significantly lower in the pandemic group than those in the pre-pandemic group(8.1 vs 8.6, *p*: < 0.001,7.7 vs 7.9,*p*:0.013, respectively). There was no significant difference between the body mass index standard deviation scores (BMI-SDS) values of the groups (0.57 vs 0.51, *p*:0.430). The initiation rate of pubertal suppression therapy at the time of presentation was significantly higher in the pandemic group compared to that of the pre-pandemic group (7.7%vs 27.5%), and in groups-2 & 3 compared to group-1, during follow-up (20%&44%vs 8%).

**Conclusion:**

Our research showed that the onset of puberty occurred earlier in the pandemic period compared to the previous year, and the need for pubertal suppression therapy increased during the pandemic.

## Implications and contribution

During the Coronavirus-19 disease (Covid-19) Pandemic, the onset of puberty in females appears to occur earlier compared to pre-pandemic period. The need for pubertal suppression treatment was increased in the era of the pandemic. However, there is still a need to clarify the underlying causes to take preventive actions in the future.

## Background

To mitigate the effects of the Covid-19 pandemic which has been impacting the whole world since January 2020, closure measures have been implemented across the world. Due to lengthy periods of school closure, restrictions on activities, and changes in diet and sleep patterns, an increase in the frequency of obesity in children is to be expected [1]. The first Covid-19 case in Turkey was reported on March 9, 2020 and shortly afterwards, on March 12, schools were closed nationwide. Subsequently, a lock-down was implemented for people younger than 20 years old between 4^th^ April and 15^th^ June [2]. During the lock-down, children were not only out of school, but they also faced severe restrictions to their daily physical routines, and in this period of inactivity, it was inevitable that there would be an increase in screen-time. When all these contributing factors are combined, it is not difficult to predict that the situation could cause rapid weight gain.

Previous studies have shown that weight gain in children is faster during the summer periods when schools are closed [3–5]. Von Hippel et al. monitored the weight increase patterns of school-age children for 3 years and showed that increases in the frequency of obesity and being overweight occurred only during summer holidays [3]. In very recent research published from Italy, it was shown that adiposity in children increased significantly during the pandemic [6]. In addition, two other studies from Italy revealed a decrease in the age of pubertal onset and an acceleration in the tempo of puberty [7, 8]. While obesity is most frequently purported as being the greatest risk factor in terms of affecting the physiology of puberty, there are also findings which suggest that physical inactivity, prolonged screen time, changes in sleeping patterns, and psychological factors also contribute directly to the problem [9, 10].

In Turkey, it was recognized that the number of children who were brought to pediatric endocrinology outpatient clinics due to early puberty increased during and just after the lock-down (unpublished anecdotal data). We also observed that, unlike in the cases of precocious puberty in previous years, these cases had markedly accelerated puberty and desynchronization between the findings of puberty. In this study, the aim was to compare the anthropometric measurements, pubertal stages, and pubertal progression rates of girls referred for early puberty in the first 6 months of the pandemic with those of girls referred in the year prior to the first Covid case (March 9, 2020).

### Materials and methods

The medical records of girls who had been referred to the pediatric endocrinology clinics of Koç University Hospital, Yeditepe University Hospital, University of Health Sciences, Umraniye Training and Research Hospital, University of Health Sciences Turkey, Diyarbakir Gazi Yasargil Training and Research Hospital, and Erzurum Regional Training and Research Hospital to be evaluated for precocious puberty were evaluated retrospectively. The participants were classified into two groups according to the time of referral—those who had presented before the pandemic (pre-pandemic group) and those who had presented during the pandemic period (pandemic group). The pre-pandemic group consisted of girls referred between March 9, 2019, and March 9, 2020, and the pandemic group between March 9, 2020, and September 9, 2020. All cases with non-idiopathic central precocious puberty (CPP), isolated premature adrenarche, and peripheral precocious puberty were excluded. The anthropometric findings, pubertal stages, and laboratory findings of the children in both groups were compared at the time of referral.

The participants with follow-up data were divided into 3 groups: those whose first admission and follow-up visit were before the pandemic were classified as group 1, those whose first admission was before the pandemic and whose follow-up visit was during pandemic were classified as group 2, and those whose first admission and follow-up visit were during the pandemic were classified as group 3. Their results of anthropometric measurements, pubertal stages, and rate of pubertal progression were recorded. The participants who had undergone gonadotrophin-releasing hormone (GnRH) analogue therapy in their first presentation were not included in this three-group analysis.

All participants underwent a physical examination and auxological evaluation. Bodyweight was measured using a digital body weight scale, and height was measured in the standing position with a wall-mounted stadiometer. Height, weight, body mass index (BMI), pubertal stage and rate of pubertal progression were recorded. BMI was calculated by dividing the patient’s weight in kilograms by the square of their height in meters. Standard deviation scores for height and BMI were calculated using an online calculator (www.ceddcozum.com) which was developed by the Turkish Pediatric Endocrinology and Diabetes Society [11]. Pubertal development was classified according to the Marshall and Tanner criteria [12]. The age of pubertal onset was defined as the age at durable Tanner B2 stage, as confirmed by clinical evaluation by a pediatric endocrinologist.

The clinical data (e.g., age at diagnosis, personal and family history of major diseases, family history of CPP), laboratory data (follicle-stimulating hormone (FSH), luteinizing hormone (LH), estradiol (E2) and gonadotropin releasing hormone (GnRH) stimulation test results), and radiological data including pelvic ultrasonography and bone age (BA) assessment were recorded. Bone age was assigned by a pediatric endocrinologist using the method of Greulich and Pyle and a pelvic ultrasonography was performed by an experienced radiologist in the local center. The data on screen time (time spent watching TV) + computer time (time spent using a computer, computer game, or internet) and physical activity of patients who had been referred during the pandemic were obtained from parents.

Precocious puberty in girls was defined as the breast development with or without axillary/pubic hair before the age of 8. A baseline LH level of > 1.1 IU/L together with pubertal signs, or a GnRH-stimulated peak LH level of > 5 IU/L with a stimulated LH/FSH ratio of > 1.0 were considered as “CPP”. Pubertal onset between 8 and 9 years of age, or presence of breasts with or without pubic hair before 8 years but a prepubertal GnRH test, were considered as “early pubertal variants”. Accelerated pubertal development was defined as a rate of progression from Tanner stage II to a further stage in less than 6 months.

All analyses were conducted using SPSS version 26 (IBM SPSS Statistics for Windows, Version 26.0. IBM Corp, Armonk, NY, USA). The frequencies and percentages represented the descriptive statistics for the categorical variables. For the continuous variables, mean and $\pm$ standard deviation(SD) values were used if the variables had a normal distribution and median values were used if the variables did not have a normal distribution. The intergroup comparison analyses were performed using the student-t and the Mann–Whitney U and Kruskal Wallis tests depending on the distribution of the analyzed variable; the comparison of categorical variables was conducted using the chi-square test. A p-value of < 0.05 was considered statistically significant.

## Results

The clinical and laboratory data of 359 females who had been referred for evaluation for precocious puberty were analyzed. Of them, 214 had presented before the pandemic, and 145 had presented during the pandemic. The demographic, clinical, and laboratory features of these two main groups were compared (Table 1). The mean age of pubertal onset was significantly lower in the pandemic group compared to the pre-pandemic group (7.7 ± 1 years vs 7.9 ± 0.9 same period/year, *p*:0.013). Parallel to this, the age at presentation of those who had presented in the pandemic was lower than those who had presented before the pandemic (8.1 ± 1.1 years vs 8.6 ± 1.2 years, *p* < 0.001). There was no significant difference in anthropometric measurements between the pandemic and the pre-pandemic groups in terms of height SDS, weight SDS and BMI-SDS. The rate of obesity was similar in these two groups. The pubertal status in the groups was similar, with a Tanner stage 2 frequency of about 60% in both groups. The difference between bone and chronological age was also similar between the two groups. However, the number of the CPP cases in the pandemic group (42 patients/6 months) was significantly higher than those in the pre-pandemic group (19 patients/12 months) (*p* < 0.001). Similarly, the ratio of those who had undergone pubertal suppression therapy at presentation during the pandemic was significantly higher than those who had presented before the pandemic. Data regarding daily screen time were only available in the pandemic group. Based on the reports from the parents, screen time had increased significantly during the pandemic compared to the pre-pandemic period (4.1 vs 2.6 h/day).

**Table 1:** The characteristics of the study groups

	**Pre-pandemic**	**Pandemic**	***P***
**Patients**	214	145	-
**Chronological age at B2 (as referred by parents) (yr)**	7.9 ± 0.9	7.7 ± 1	0.013
**Chronological age at diagnosis (yr)**	8.6 ± 1.2	8.1 ± 8.1	** < 0.001**
**Height SDS**	0.75 ± 1.02	0.63 ± 1.15	0.31
**Weight SDS**	0.76 ± 0.93	0.74 ± 1.02	0.89
**BMI SDS**	0.57 ± 0.86	0.51 ± 0.92	0.57
**BMI-Status-***n* (%)			
** Obese**	17 (8)	14(9.7)	0.430
** Overweight**	48(22.6)	24 (16.6)	0.180
** Normal**	144(67.9)	103(71)	0.306
** Underweight**	3(1.4)	4(2.8)	0.300
**Tanner stage at diagnosis-***n*(%)			
** T II**	126(59.2)	88(60.7)	0.217
** T III**	61(28.59)	45(31)	0.667
** T IV**	20(9.3)	12(8.3)	0.697
** T V**	6(2.8)		0.085
**Bone age (yr)**	9.4 ± 1.42	9.14 ± 1.48	0.18
**Bone age-chronological age (yr)**	0.94 ± 1.02	1.1 ± 0.91	0.182
**Basal LH (IU/L)**	0.74 ± 1.6	0.78 ± 1.3	0.489
**Basal estradiol (pg/ml)**	19.5 ± 14	16.5 ± 13	0.08
**Peak LH at LHRH stimulation test (IU/L)**	6.6 ± 3.9	6.5 ± 4.7	0.6
**Uterine length (mm)**	34.5 ± 9.6	39 ± 33	0.51
**Ovarian volume (cm**^**3**^**)**	3 ± 2.3	2.4 ± 1.4	0.056
**Diagnosis at the time of presentation**			
** Central Precocious Puberty,** *n* (%)	19 (8)	42 (29)	**< 0.001**
** Early pubertal variant,** ***n*** (%)	195 (91)	103 (71)	<0.001
**GnRH analogue treatment at presentation-** ***n***(%)	16(7.7)	40 (27.5)	** < 0.001**
**Screen time (h/day)** **(*n***:35)	2.6	4.1	** < 0.001**
**Physical activity (h/day)** **(*n***:35)	2.7	2	0.81

The data of 99 patients who had medical records of their follow-up were analyzed separately in three groups (Table 2). “Group 1” consisted of 60 girls whose first presentation and follow-up visits had taken place before the pandemic, “group 2” consisted of 21 girls whose first presentation had taken place before the pandemic, but whose follow-up visit had taken place during the pandemic and, “group 3” consisted of 18 girls whose first presentation and follow-up visits had taken place during the pandemic. The mean age at presentation of girls in group 3 was significantly lower than those in groups 1 and 2 (7.63 vs 8.4 & 8.1 years, *p*:0.04). The mean duration between presentation and follow-up visit was similar in the 3 groups (0.45 years in group 1, 0.46 years in group 2, and 0.34 years in group 3). Although the auxological parameters for height SDS, BMI SDS, and bone age SDS were not statistically different between the groups, BMI SDS increased significantly in group 2 during follow up in the pandemic (*p* < 0.05), accordingly the mean “delta BMI-SDS” was significantly higher in group 2 when compared to groups 1 and 3 (0.21 vs -0.02 & -0.06, *p*: 0.004). The number of patients who started to receive treatment with a GnRH analogue on their second visit was significantly higher in groups 2 and 3 compared to group 1.

**Table 2:** Anthropometric measurements and change in pubertal status in follow-up, a comparison of the groups

	**Group 1 (*n*:60)**		**Group 2 (*n*: 21)**		**Group 3 (*n*:18)**	
	Visit 1-before pandemic	Visit 2-before pandemic	Visit 1-before pandemic	Visit 2-pandemic	Visit 1-pandemic	Visit 2-pandemic
**Chronological age (yr)**	8.4	8.8	8.1	8.6	7.63*	**7.9**^**†**^
**Duration between the two visits (yr)**	-	0.45	-	0.46	-	0.34
**BMI SDS**	0.67	0.59	0.24	0.48	0.29	0.13
**Height SDS**	0.71	0.8	0.89	1.2	0.98	0.87
**Delta BMI-SDS (median)**	-	-0.02	-	**0.21**^‡^	-	-0.06
**Delta height SDS (median)**	-	0.14	-	0.24	-	0.08
**Tanner stage (%)**						
** T II**	61.7	41.7	71.4	38.1	66.7	27.8
** T III**	31.7	33.3	14.3	33.3	22.2	55.6
** T IV**	5	10	4.8	9.5	11.1	11.1
** T V**	1.7	5	4.8	4.8	0	0
**Change in pubertal stage during follow-up**						
**No change (%)**	-	67%	-	57%	-	61%
** + 1 stage (%)**	-	32%	-	38%	-	39%
** + 2 stages (%)**	-	1%	-	5%	-	0
**GnRH analogue therapy in follow-up n (%)**	-	**5(8)**^**§**^	-	4(20)	-	8(44)
**Basal LH (median) (IU/L)**	0.1	-	0.17	-	0.25	-
**Peak LH at LHRH stimulation test (IU/L)**	5	-	8.2	-	5.6	-
**Basal estradiol (median)(pg/ml)**	19.5	-	16	-	21.5	-
**Uterine length (mm)**	35.5	-	32.5	-	38	-
**Ovarian volume (cm**^**3**^**)**	3	-	2.6	-	2.5	-
**Screen time (h/day)**	-	-	1.5	3	2.1	3.25

^*^p:0.013 (group 3 vs groups 1&2),^†^*p*:0.005 (group 3 vs groups 1&2),^‡^*p*:0.04 (group 2 vs groups 1&3),

^§^*p*:0.003 (group 1 vs groups 2&3

## Discussion

The results of this multi-center study showed that the girls who had been referred for early puberty during and after the lockdown for the COVID-19 pandemic was younger than those who presented during the year prior to the pandemic. It is interesting to note that real precocious puberty was more prevalent than pubertal variants, and the rates of treatment with GnRH analogues had increased as expected.

The first report on the accelerated pubertal tempo during pandemic was published from Italy by Stagi et al., which stated that there was an increased incidence of newly diagnosed CPP and a faster rate of pubertal progression in patients previously diagnosed during and after the lockdown period. They hypothesized that triggering environmental factors, such as weight gain and the use of electronic devices, were amplified during lockdown [7]. Following this, Verzani et al. reported their preliminary data which showed an increase in the prevalence of female cases with premature thelarche (215 patients in 2020 versus 87 patients in 2019) [8].

While it is well-known that nutrition is a factor affecting pubertal timing and a driver of sexual maturation, and that obesity is a significant intensifying factor in the development of early puberty [13, 14], in the current study, BMI SDS values were not found to be different in pre-pandemic and pandemic groups. Very recently, Acar and Ozkan from Turkey reported that the BMI-SDS values and frequency of obesity were similar in their patients diagnosed with idiopathic CPP during and before the Covid-19 pandemic lockdown, like our results [15]. Reproduction is an energy-consuming process, particularly in females, and therefore, the acquisition and maintenance of reproductive capacity are closely tied to the state of body energy reserves. Accordingly, energy dysregulation and changes in metabolic homeostasis are associated with the onset of puberty. Therefore, chronic energy deficiency such as malnutrition and anorexia nervosa causes a delay in the onset of puberty, whereas obesity and/or overnutrition, where body energy stores are excessive, cause early-onset puberty. Indeed, in the presence of a positive energy balance, mTOR is dominant in the mTOR / AMPK pathway [16]. In addition, Vazquez et al. showed that changes in SIRT1 activity play a role in accelerating puberty by increasing Kiss-1 expression. In contrast, an insufficient/negative energy balance causes inhibition of Kiss-1 expression and delays puberty [16]. In this study, although the BMI-SD scores were not significantly different in the pre-pandemic and pandemic groups, the delta BMI-SD scores were higher in girls whose first presentation was before the pandemic, but the follow-up visit during the pandemic. These findings support the results of Vazquez’s study by leading us to think the velocity of weight gain, as well as the amount of weight gain, may be related to the pubertal onset and rapid pubertal development.

Changes in sleep habits may also have played a role in the emergence of early and accelerated pubertal progression [17]. When schools are closed, children tend to go to bed very late and then wake up late, especially during the summer holidays. The sleep and reproductive axis are in a close relationship during pubertal development. Melatonin has a role in suppressing the reproductive axis during childhood. Melatonin levels were found inversely correlated with serum LH levels and lower in children with CPP than in age-matched controls [18]. Although data on the sleep characteristics of the participants was not available for the study, the daily screen time was reported to be higher compared to the pre-pandemic period in group 2 (pandemic group). Excessive exposure to television and computer games during long vacations may cause a sleep disorder vicious cycle by delaying sleep time and a decrease in melatonin levels. Exposure to simulated electromagnetic fields has been shown to decrease melatonin production in vitro and decrease plasma melatonin and its urinary metabolites [19]. There is no doubt that the pandemic caused an overall increase in screen time, not only for entertainment purposes, but also for school activities. Guo et al. reported that there had been changes in screen time, quality of sleep, and physical activity in the students in the Guangzhou region in China—the region where Covid-19 was first reported [20]. The length of screen time is also associated with reduced physical activity, contributing to the positive energy balance. However, there is a need for more comprehensive research to decide whether these changes might have effects on the timing and progression of puberty.

Other factors that are claimed as being possible causes of rapid pubertal progress are the emotional and psychological factors during pandemic, a period which was defined as “a biological disaster with a subsequent strong psychological impact” [21]. Concerns about the parents getting sick, avoidance of social environments, financial problems of family could contribute to emotional stress. Alterations in the levels of catecholamines (dopamine and norepinephrine-NE) might be a possible mechanism in the acceleration in puberty—a theory which has been shown in an animal model [22]. However, as of yet, there is no compelling evidence concerning this mechanism in humans.

The need for puberty suppressing therapy at the time of presentation was higher in the pandemic group compared to the pre-pandemic group. Similarly, the analysis of the cases with follow-up data showed that the rate of initiation of treatment in the follow up visit was lower in group 1 (whose first and follow-up visits had taken place before the pandemic) compared to the other groups. Although there was no difference in puberty progression rates, the fact that the treatment rate was higher in cases in the pandemic period was possibly related to the younger age at diagnosis and follow-up. On the other hand, it could be that the attitudes of the physicians participating in the study towards starting puberty suppressing treatment may have differed. As Kaplowitz pointed out in a review, the rate of treatment initiation in borderline cases (onset of puberty between 7 and 9 years old) has increased in recent years, however the indicators which have been suggested for the medical necessity of treatment, including psychological stress and loss in predicted adult height, have not been fully clarified [23].

The main limitation of our study was the lack of information on lifestyle changes due to the retrospective collection of data from different centers. Before the pandemic, patients with puberty disorders were not evaluated in detail in terms of changing factors in daily lifestyle. However, the strengths of our study are that it was multicenter and used a comparison of follow-up data, albeit over a short-term period.

## Conclusions

Our research has shown that the onset of puberty was earlier in the pandemic period compared to the previous year, and the need for pubertal suppression therapy increased in this period. The lesson we have learned from this study was that we should follow up puberty more carefully and question environmental factors in more detail in all children of peripubertal age. However, there is still a need to clarify the underlying causes which, along with elucidating underlying mechanisms, would make it possible to take preventive actions in the future. Long-term comprehensive research is also needed to understand whether the acceleration in pubertal tempo will continue when the pandemic ends.

## Data Availability

The datasets used and/or analysed during the current study are available from the corresponding author on reasonable request.

## Abbreviations

BMI: Body mass index
COVID-19: Coronavirus-19 disease
CPP: Central precocious puberty
E2: Estradiol
FSH: Follicle-stimulating hormone
GnRH: Gonadotrophin-releasing hormone
IU/L: International unit per liter
LH: Luteinizing hormone
LHRH: LH-releasing hormone
SD: Standard deviation
SDS: Standard deviation scores
